# Estimating the Life Course of Influenza A(H3N2) Antibody Responses from Cross-Sectional Data

**DOI:** 10.1371/journal.pbio.1002082

**Published:** 2015-03-03

**Authors:** Adam J. Kucharski, Justin Lessler, Jonathan M. Read, Huachen Zhu, Chao Qiang Jiang, Yi Guan, Derek A. T. Cummings, Steven Riley

**Affiliations:** 1 Department of Infectious Disease Epidemiology, London School of Hygiene & Tropical Medicine, London, United Kingdom; 2 MRC Centre for Outbreak Analysis and Modelling, Department of Infectious Disease Epidemiology, School of Public Health, Imperial College London, London, United Kingdom; 3 Department of Epidemiology, Johns Hopkins Bloomberg School of Public Health, Baltimore, Maryland, United States of America; 4 Department of Epidemiology and Population Health, Institute of Infection and Global Health, Faculty of Health and Life Sciences, University of Liverpool, Liverpool, United Kingdom; 5 International Institute of Infection and Immunity, Shantou University Medical College, Shantou, Guangdong, China; 6 State Key Laboratory of Emerging Infectious Diseases and Centre of Influenza Research, University of Hong Kong, Hong Kong SAR, China; 7 Guangzhou No. 12 Hospital, Guangzhou, Guangdong, China; The Pennsylvania State University, UNITED STATES

## Abstract

The immunity of a host population against specific influenza A strains can influence a number of important biological processes, from the emergence of new virus strains to the effectiveness of vaccination programmes. However, the development of an individual’s long-lived antibody response to influenza A over the course of a lifetime remains poorly understood. Accurately describing this immunological process requires a fundamental understanding of how the mechanisms of boosting and cross-reactivity respond to repeated infections. Establishing the contribution of such mechanisms to antibody titres remains challenging because the aggregate effect of immune responses over a lifetime are rarely observed directly. To uncover the aggregate effect of multiple influenza infections, we developed a mechanistic model capturing both past infections and subsequent antibody responses. We estimated parameters of the model using cross-sectional antibody titres to nine different strains spanning 40 years of circulation of influenza A(H3N2) in southern China. We found that “antigenic seniority” and quickly decaying cross-reactivity were important components of the immune response, suggesting that the order in which individuals were infected with influenza strains shaped observed neutralisation titres to a particular virus. We also obtained estimates of the frequency and age distribution of influenza infection, which indicate that although infections became less frequent as individuals progressed through childhood and young adulthood, they occurred at similar rates for individuals above age 30 y. By establishing what are likely to be important mechanisms driving epochal trends in population immunity, we also identified key directions for future studies. In particular, our results highlight the need for longitudinal samples that are tested against multiple historical strains. This could lead to a better understanding of how, over the course of a lifetime, fast, transient antibody dynamics combine with the longer-term immune responses considered here.

## Abstract 2

A combination of cross-sectional serological data from Southern China and mathematical modeling reveals key aspects of the human immune system's response to seasonal influenza infection.

## Author Summary

Host immunity against seasonal influenza viruses influences the emergence of new virus strains, the size and severity of “flu” epidemics, and the effectiveness of vaccination programmes. However, the specific factors that shape the immune response of a single human to a particular strain are little understood because individual infections and the development of immunity over a lifetime in that person are rarely observed directly. To determine the aggregate effect of a lifetime of influenza infections on host immunity, we developed a mathematical model that captures the specific strains with which an individual has been infected and for the corresponding antibody response, the relative contribution of boosting, cross-reactivity, and antigenic seniority to its neutralising ability. Combining the model with data from a survey in southern China that examined antibody levels against nine different influenza strains from 1968 to 2009, we revealed key components of the immune response to influenza virus infection, and obtained estimates of the frequency of influenza infection and the ages at which infection occurred. Our results suggest that “antigenic seniority”, whereby strains encountered earlier in life gain more “senior” positions in the immune response, and short-lived cross-reactivity between different strains are important components of the immune response and, therefore, could shape the evolution and emergence of influenza viruses.

## Introduction

The immunity of a host population against specific influenza A strains can influence a number of important biological processes. It can affect the emergence of new virus strains, and hence shape the evolution of the disease [1,2]. It can also influence the size and severity of a pandemic [3–6], and the effectiveness of vaccination programmes [7].

There are two main ways to measure the adaptive immune response against influenza viruses [8]. In microneutralisation assays, a mixture of virus and diluted serum is used to infect cell cultures; the titre is the highest dilution for which virus infection is blocked. Microneutralisation titres therefore measure the overall neutralising antibody response. Such a response can include several components. Some antibodies are specific to antigenic sites on the globular head of the haemagglutinin (HA) surface protein. These sites are highly variable: the HA undergoes frequent mutation, enabling the virus to escape existing antibody responses [9]. There is also evidence that antibodies target conserved epitopes on the stalk of the HA protein or the neuraminidase (NA) surface protein [10–12]. Alternatively, haemagglutination inhibition (HAI) assays measure the extent to which antibodies inhibit binding of the HA protein to red blood cells. Whereas microneutralisation titres likely capture more of the total antibody response, the HAI assay is a more sensitive measure of antibodies that are specific for antigenic sites on the head of the HA protein [13].

The ability of human sera to neutralise current and historical influenza strains exhibits substantial variation between individuals and with age [10,13–15]. These patterns are likely to be influenced by a number of factors. First, neutralisation titres to a particular strain depend on the immune response following exposure to that virus: after infection or vaccination, the immune response to a particular virus can be boosted [5]. Although the initial response may decay to a lower level after a short period of time [16,17], there is evidence that the subsequent level of response can persist for several decades [18].

Observed titres can also depend on the order and number of influenza infections. Francis [14] coined the term “original antigenic sin” to describe the phenomenon by which HAI titres to the first influenza infection of a lifetime were apparently higher than titres to other strains. Upon subsequent infection, it has been suggested that the original response can be enhanced [14,19–21] and the antibody response to the new strain reduced [22–26]; for original antigenic sin to occur, there is evidence that the original and new strain must be antigenically related [26,27]. Recent work has refined the original antigenic sin hypothesis, proposing that serological patterns should be described in terms of “antigenic seniority” [13,15]. As with original antigenic sin, the primary infection gains the most “senior” position in the immune response, but—as a key refinement to the original sin hypothesis—the hierarchy of responses continues with each subsequent infection, as each strain takes a less senior position in the response.

As well as boosting and original antigenic sin/antigenic seniority, observed serological responses can also depend on cross-reactivity between strains and temporal waning of responses. Even if hosts have not been exposed to a given strain, they can have a raised titre against the virus if the test strain is similar to those already encountered [5,18]. Establishing the contribution of different mechanisms to neutralisation titres remains challenging, however, because the aggregate effect of immune responses over a lifetime are rarely observed directly [13,24]. Moreover, observed titres not only depend on the relationship between infection and immune response: they are also influenced by the specific strains a host has been infected with. It has been suggested that certain age groups are infected more often than others [28,29], but the true frequency of influenza infection cannot be easily measured [30].

To explore the effects of past infections and subsequent immune responses on observed microneutralisation titres, we fitted a mechanistic model of within-host serological dynamics to data from a cross-sectional survey based in southern China [31]. In the study, 151 individuals’ sera were tested against a panel of nine different influenza A(H3N2) strains isolated between 1968 and 2008. Six of these strains corresponded to representative viruses from every second “antigenic cluster” that appeared between 1968 and 2003; in total there were 11 such clusters of antigenically similar strains during this period [32]. The other three test viruses were strains that circulated in southern China between 2003 and 2008.

We used the mechanistic model to assess the relative contribution of boosting, cross-reactivity, and antigenic seniority to observed neutralisation titres, and estimated key immunological parameters. We also estimated which specific strains each individual had been infected with, and hence assembled infection histories for each individual in the study population. This made it possible to calculate the frequency of infection for influenza A(H3N2) in the host population, and to establish how the infection rate varied with age.

## Materials and Methods

### Ethics Statement

Study protocols and instruments were approved by the following institutional review boards: Johns Hopkins Bloomberg School of Public Health, University of Liverpool, University of Hong Kong, Guangzhou No. 12 Hospital, and Shantou University. Written informed consent was obtained from all participants over 12 y of age, and verbal assent was obtained from participants 12 y of age or younger. Written permission of a legally authorised representative was obtained for all participants under the age of 18 y.

### Data

Participants were recruited from five study locations, with 20 households randomly selected in each location (further details given in Lessler et al. [31]). Participants’ sera were tested against nine representative influenza A(H3N2) strains using a virus neutralisation assay. The strains included six vaccine strains: A/Hong Kong/1/1968, A/Victoria/3/1975, A/Bangkok/1/1979, A/Beijing/353/1989, A/Wuhan/359/1995, and A/Fujian/411/2002. Three strains that circulated in southern China in the years preceding the study were also tested: A/Shantou/90/2003, A/Shantou/806/2005, and A/Shantou/904/2008. Titres were measured using serial 2-fold dilutions from 1:10 to 1:1,280 in duplicate. In our analysis, we represented the results in terms of log neutralisation titres. A log titre of *c* corresponded to a dilution of 10 × 2^(*c*−1)^. Hence, there were nine possible log titres: the lowest was 0, which corresponded to a dilution <1:10; the highest was 8, which corresponded to a dilution of 1:1,280.

### Model of Serological Dynamics

We took an “epochal” view of infection [32,33], with individuals either infected or not during each antigenic epoch; we assumed there were 14 such epochs between 1968 and 2008. We modelled serological titres by assuming that the mean neutralisation titre to a specific strain depended on both individual infection history and a combination of serological mechanisms. We considered four specific mechanisms: boosting from infection with the test strain, cross-reactivity from antigenically similar strains, boosting of earlier responses as a result of subsequent infection, and suppression of subsequent responses as a result of prior immunity. The final two mechanisms have been suggested as potential explanations for observed patterns of antigenic seniority [13,15].

**Fig 1:**
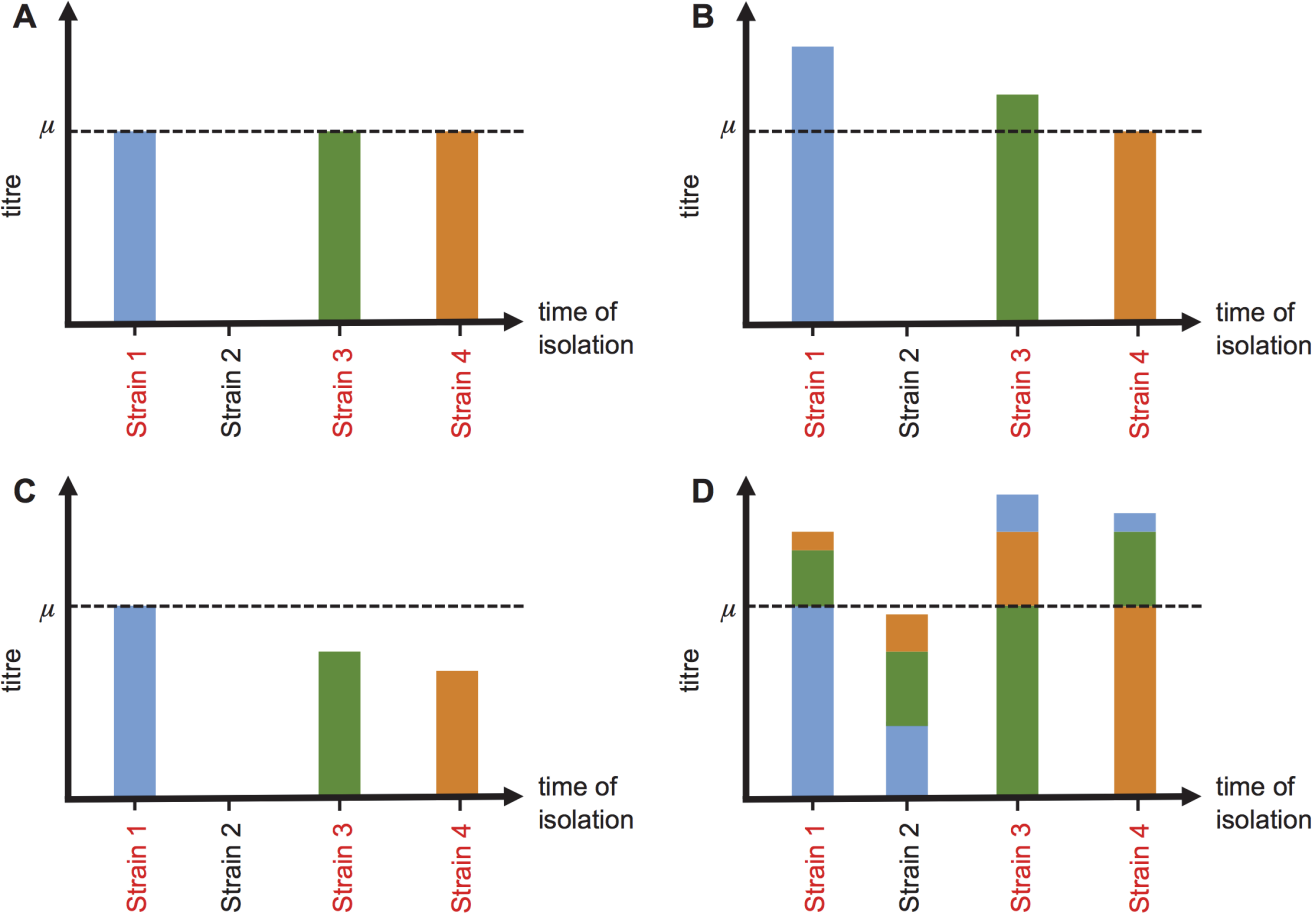
Schematic of mechanisms that shape observed titres in the model. (A) Simple boosting. In the absence of cross-reactivity and antigenic seniority, if an individual had been infected with a particular strain, they exhibited a fixed response to that strain equal to μ. This was controlled by a single parameter in the model. In the figure, strains are sorted by date of isolation, with serological samples taken in present day. Strains the host has been infected with are shown in red; coloured bars show the magnitude of observed log titre as a result of past infection with each strain. (B) Boosting of prior responses via antigenic seniority. Infections boosted observed titres to earlier infecting strains by a certain scaling factor, controlled by the parameter τ1. The magnitude of titre to a particular strain therefore depends on the number of infections that occurred after infection with that strain. (C) Suppression of new responses via antigenic seniority. The response to each strain was reduced as a result of immunity generated by previous infections. This reduction was controlled by the parameter τ2. The titre to a particular strain therefore depended on the number of infections that occurred before that strain circulated. (D) Cross-reactivity. In the absence of antigenic seniority, the observed titre to a test strain depended on the response as a result of infection with that strain, plus cross-reactive responses from infection with other strains. These cross-reactive responses decreased with the distance (measured in years) between each infection and the test strain. Strains that circulated further from the test strain in time contributed less to the observed response.

We included the four main mechanisms in the model as follows. Suppose an individual has an infection history that consists of a set of strains *X* (note that we do not distinguish between live infection and vaccination in the model). We assumed that if an individual had been infected with only one strain, they would exhibit a fixed log titre against that strain, controlled by a single parameter, μ. In the absence of antigenic seniority or cross-reactivity, the individual would therefore have titre equal to μ for every strain in their infection history, and zero for all other strains (Fig. 1A). However, if the individual had been infected with more than one strain, titres against earlier strains could be higher than those against later strains as a result of antigenic seniority.

Two mechanisms have been proposed to explain observed patterns of antigenic seniority: previous responses might be boosted as a result of subsequent infections, or subsequent responses might be reduced as a result of previous immunity [15]. To evaluate the contribution of these two mechanisms, we specified the model so that—depending on parameter values—both, one, or neither mechanism could contribute to measured titres. During the fitting process, model outputs could therefore be compared to observed serological data to establish which mechanism(s) were most plausible given the data.

First, we assumed each infection could boost titres against strains encountered previously by a parameter τ_1_ (Fig. 1B). Hence the titre μ was scaled by a factor *s*_1_(*X*, *j*) = (1 + *τ*_1_)^|*X*|−*N*_*j*_^ where *N*_*j*_ is the number of the strain in the infection history (i.e., the first strain is 1, the second is 2, etc.) and |*X*| is the total number of infections. If τ_1_ = 0, then there was no boosting as a result of subsequent infection. This mechanism, in which observed titres to a particular strain depended on the number of subsequent infections, was also proposed by Miller et al. [13] following a longitudinal study of influenza A infections.

Second, we assumed prior immunity could reduce observed titres against strains encountered later in life (Fig. 1C). The titre against a particular strain would therefore be scaled by a factor $s_{2}\left(X,j\right)=e^{-\tau_{2}\left(N_{j}-1\right)}$. Here, observed titres to each strain depended on how many infections had occurred previously. When τ_2_ = 0, prior infections did not lead to reduced responses against later strains. When τ_2_ was large, the formulation was equivalent to a model of original antigenic sin, in which immunity from the primary infection suppressed all subsequent responses [28,34].

Finally, we incorporated cross-reactivity by assuming that mean titre against a specific strain was equal to the sum of cross-reactive responses to all strains in an individual’s infection history. We assumed that the contribution made by each strain depended on the temporal distance between the strain in the infection history and the test strain (Fig. 1D). The level of cross-reaction between a test strain *j* and infecting strain *m* was given by *d*(*j*, *m*) = *e* ^−*σ*|*t_m_*−*t_j_*|^, where *|t*_*m*_ *− t*_*j*_ *|* was the number of years between strains *j* and *m*, and σ was a parameter to be fitted. If σ was large, it was equivalent to having no cross-reactivity between strains.

To combine the four mechanisms in the model, we assumed that the log titre individual *i* has against a strain *j* was Poisson distributed with the following mean:

$$\lambda_{ij}=\mu \sum_{m\in X}d(j,m)s_{1}(X,m)s_{2}(X,m)$$ (1)

As *d*(*j*, *m*), *s*_1_(*X*, *m*), or *s*_2_(*X*, *m*) could equal 1 for certain parameter values, the model was capable of omitting certain mechanisms if necessary.

We also accounted for potential observation error by assuming that there was a uniform probability of observing a titre different to the true one. Hence, the likelihood of observing titre *c*_*j*_ against test strain *j* was equal to the sum over all possible true titres:

$$L\left(c_{j}\right)=\mu \sum_{k}P\left(\text{true titre is}\;k\right)\times P\left(\text{observe}\;c_{j}\;|\;\text{true titre is}\;k\right)$$ (2)

We estimated model parameters using Markov chain Monte Carlo (details in S1 Text; dataset and model outputs in S1 Data).

As a sensitivity analysis, we also included the waning of antibody responses in our model. This was achieved by modifying Equation 1:

$$\lambda_{ij}=\mu \sum_{m\in X}d\left(j,m\right)\;s_{1}\left(X,m\right)\;s_{2}\left(X,m\right)e^{-{\mathrm{wt}}_{m}}$$ (3)

where *w* was a waning parameter that we fixed. The formulation meant that waning reduced titres to strains in the infection history by a factor *e* ^*−w*^ per year. If *w* = 0, then we recovered the model given by Equation 1.

## Results

**Fig 2:**
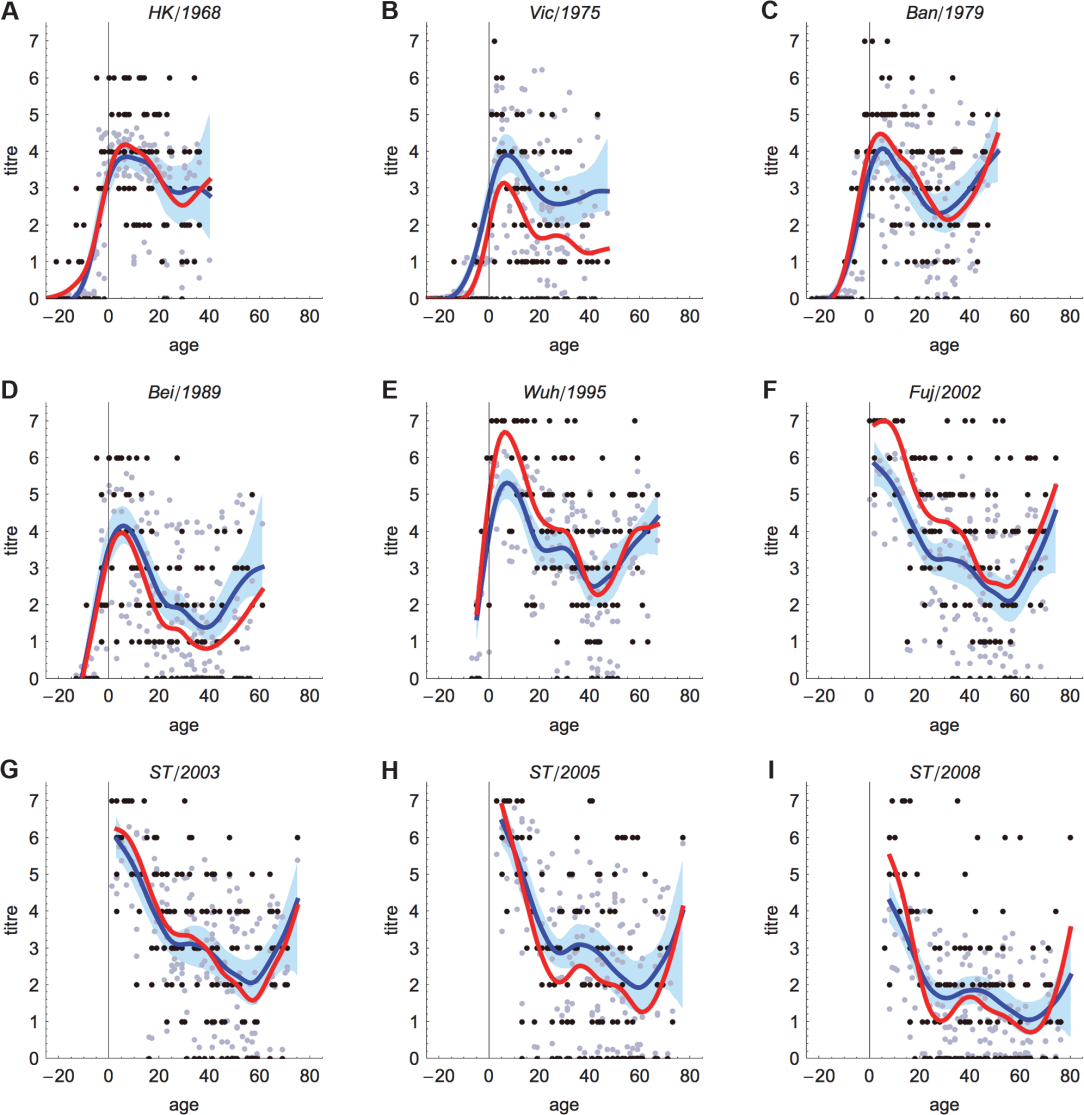
Estimated titres by strain and participant age. Black points show observed titre against that strain for each participant. Grey points show model estimates. Red line is spline fitted to the data; blue line shows spline fitted to the model estimates, with the 95% confidence interval given by the shaded region. (A–I) Results for each of the nine test strains. Parameters in the model are taken from the maximum a posteriori probability estimates. HK, Hong Kong; ST, Shantou.

The model captured the observed age distribution of titres for each of the nine test strains. Fig. 2 shows that when splines were fitted to the data (red line) and the model (blue line), there was a similar pattern with age. For time periods that were well represented by test strains in the data, such as 2003–2008, the model captured both the average pattern of titres with age and the variability in titre levels for strains in that period (Fig. 2G–I). When test strains circulated further away in time from neighbouring data points, the estimates did not match the magnitude of titre in the serological data as closely. For example, the model overestimated titre levels against A/Victoria/1975 (Fig. 2B), which was two antigenic clusters from neighbouring test strains. However, even for time periods that are less well represented in the test strains, such as 1968–1989, the model captured the correct average trend for titre levels. While both the model and data generally exhibited more variation in titre levels for more recent strains (S1 Fig.), across all strains, 87% of model estimates were within two dilutions of the observed titre (S2 Fig.).

We found evidence that antigenic seniority and quickly decaying cross-reactivity were important components of the immune response, and obtained measurements for the immunological processes outlined in Fig. 1. Parameter estimates are shown in Table 1. The boosting parameter suggests that primary infection resulted in a log neutralisation titre of around 3 (corresponding to a dilution of 1:40). Our estimate for the exponential decay in cross-reactivity with time was 0.29, suggesting that strains circulating 2.4 y apart had only 50% cross-reactivity. The antigenic seniority parameter controlling suppression of subsequent responses was 0.06, which implies that the response to each new infection was scaled by a factor 0.94 compared with the response to the previous infecting strain. In contrast, we estimated the parameter that controlled boosting of prior responses to be zero.

**Table 1:** Parameter estimates.

Parameter	Definition	Estimate (95% CI)
μ	Primary boosting	3.02 (2.66–3.42)
ε	Measurement error	0.00 (0.00–0.03)
σ	Cross-reactivity	0.29 (0.25–0.33)
τ_1_	Antigenic seniority (boost prior response)	0.00 (0.00–0.01)
τ_2_	Antigenic seniority (suppress new response)	0.06 (0.02–0.09)

To further investigate our finding that boosting from antigenic seniority was not required in the model, we compared the observed titre against the earliest strain in each individual’s infection history with their estimated total number of infections. If in reality subsequent infections boost earlier responses, we would expect the titre against the earliest strain to be larger for individuals who have been infected with numerous strains. However, we did not find a significant correlation between the two variables, suggesting that boosting from later infections had little effect on observed neutralisation titres to the first strain (S3 Fig.).

As there may be a trade-off between the short-term and long-term dynamics of influenza infection, we also examined how our estimate for boosting from antigenic seniority changed if we assumed that titres could wane over time after the initial infection [35–38]. First we tested whether the boosting parameter τ_1_ could be robustly measured if we included waning in the model. We found that there was a trade-off between the two processes in the model: a high degree of boosting was balanced by a larger amount of waning. This suggested that these two mechanisms were not distinguishable given our cross-sectional data (S4 Fig.). We therefore fixed the degree of waning, and estimated the other parameters. Our estimates for cross-reactivity and measurement error remained consistent. We still found evidence for suppression of response via antigenic seniority, and if we assumed an increased amount of waning per year, we obtained a non-zero estimate for antigenic seniority boosting (S1 Table).

In addition, we examined whether broadly cross-reactive antibodies might contribute to observed titres, as has previously been observed during influenza infection [12,39,40]. We extended the original model described in Equations 1 and 2 to incorporate a fixed amount of broad cross-reaction between distant strains (S5 Fig.; details in S1 Text). However, when we fitted this model to data, the parameter estimate for broad cross-reactivity was zero (S2 Table). We therefore recovered the original model formulation and parameter estimates, which suggested that broad cross-reactivity was not required to reproduce the observed data.

**Fig 3:**
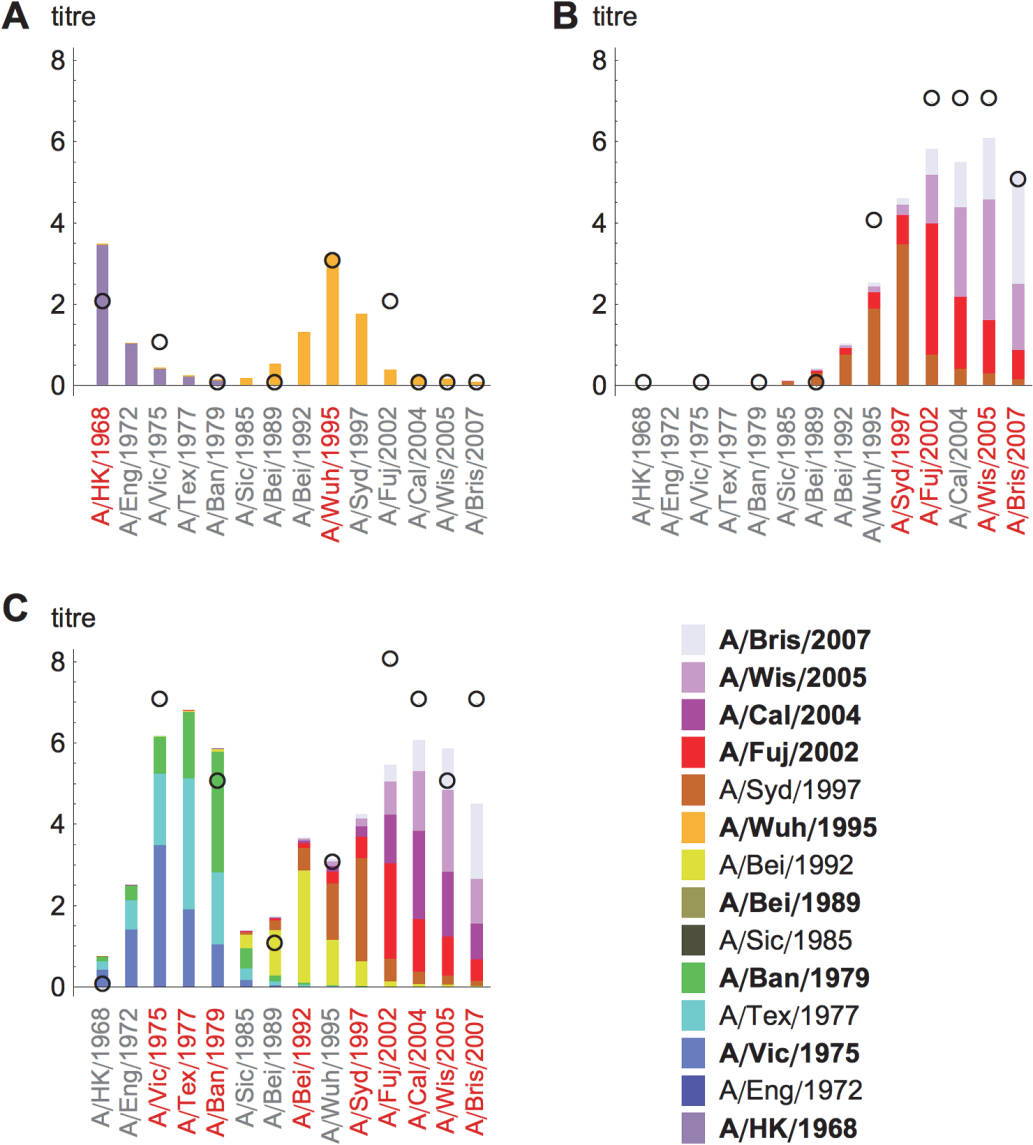
Characteristic patterns from different immune mechanisms. (A) Model titres for participant aged 64 y. Parameters in the model are taken from the maximum a posteriori probability estimate. Circles give observed titres; bars give predicted titres and are coloured by the contribution to immunity from each strain the individual was infected with (infections are indicated by strains in red on the *x*-axis). Clusters for which there are test strains are shown in bold. Here, predicted titres are predominantly the result of boosting, with little contribution from cross-reactivity. (B) Model titres for participant aged 12 y. Predicted titres for later strains were the sum of contributions from boosting with the test strain and cross-reactivity from related ones. (C) Model titres for participant aged 36 y. With each strain encountered, antigenic seniority reduced boosting to subsequent infections: the coloured bars generated by the infecting strain decrease in size as the number of infections increases.

We were able to better understand how the model reproduced observed titre values for individual people by considering specific examples. If a participant was infected with a small number of strains in the model, observed titres were predominantly the result of boosting (Fig. 3A). The sharp decay in cross-reactivity in the model meant that a low titre was produced against strains that circulated a number of years before or after the infecting strain. When participants were infected with several similar strains, the expected titre was the sum of contributions from boosting with the test strain and cross-reactivity from related ones (Fig. 3B). Cross-reactivity also led to a high titre against nearby strains, even if the strains were not in the infection history. However, antigenic seniority meant that the contribution to boosting from infection declined with each strain encountered (S6 Fig.). As a result, much of the expected titre against the first strain came from boosting with that strain, whereas titres to later strains have a larger contribution from cross-reactivity (Fig. 3C). Model residuals for these three selected examples were representative of the study population (S7 Fig.).

**Fig 4:**
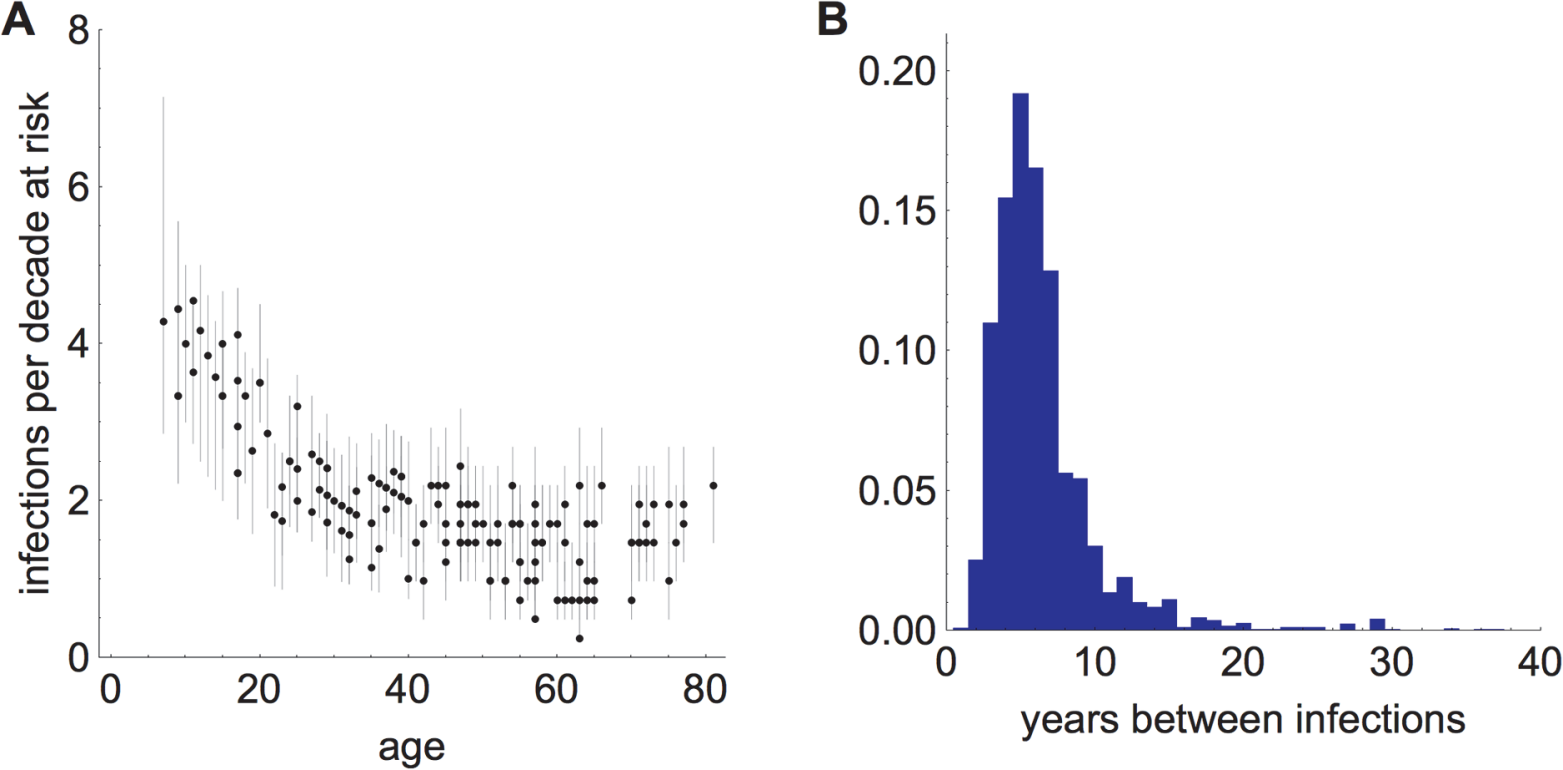
Frequency of influenza infection. (A) Number of infections per decade at risk. For each participant, this is calculated by dividing the estimated total number of infections by whichever value is smaller: participant age or 41 (total years between appearance of A(H3N2) in 1968 and test in 2009). Points give median of the posterior distribution; vertical lines show 95% credible interval. (B) Distribution of time between sequential infections, conditional on having at least two infections, across all participants and strains.

Because we inferred infection histories for each individual, it was also possible to generate estimates for frequency of infection. Fig. 4A shows the number of A(H3N2) influenza infections per decade at risk, based on the estimated infection histories. The rate of infection decayed initially with age, but was relatively flat after age 30 y, implying that above a certain age, individuals were infected with similar frequency. Fig. 4B shows the distribution of time between two sequential infections, conditional on individuals’ having had at least two infections.

We also tested the ability of the model to predict unseen data. We omitted each of the nine test strains in turn, refitted the model to the remaining eight strains, and used our parameter estimates to predict the omitted data. S8 Fig. shows that although the model captured the general pattern of measured serological response for many strains, the predictive power was highest when test strains were close together in time (S8G–I Fig.).

## Discussion

**Fig 5:**
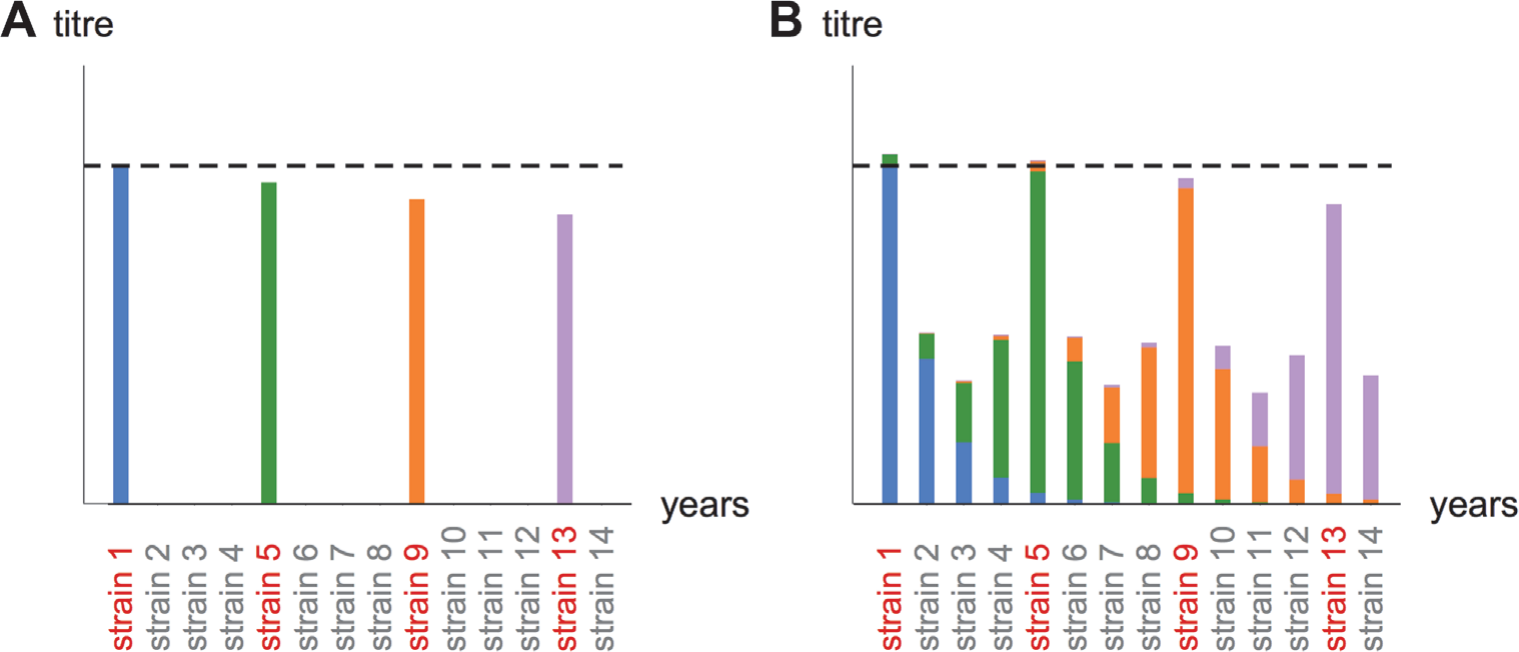
Schematic of mechanisms that shape observed titres. Our model suggests that the expected magnitude of titres that result from a sequence of infections depends on three of the four mechanisms described in Fig. 1: simple boosting, suppression of subsequent responses as a result of antigenic seniority, and cross-reaction. The contribution from infecting strains to observed titres is influenced by simple boosting and suppression via antigenic seniority (A). These contributions, as well as cross-reaction between similar strains, influence final observed titres (B). For illustrative purposes, strains here appear in 3-y-long epochs, and have circulated over a 40-y period. Strains the host has been infected with are shown in red; coloured bars show the magnitude of observed log titre as a result of past infection with each strain.

We have examined how past infections with influenza A(H3N2) strains influence observed cross-sectional neutralisation titres. We found that “antigenic seniority” and quickly decaying cross-reactivity were important components of the immune response. The order in which an individual is infected with influenza strains was therefore important in dictating observed titres to a particular virus: titres appeared to be the result of a combination of strain-specific boosting, cross-reactivity, and suppression of subsequent responses as a result of antigenic seniority (Fig. 5).

Our results emphasise the importance of understanding how currently unobserved mechanisms shape the dynamics of influenza for individuals over the course of their lifetime. Traditionally, analysis of serological data has been descriptive rather than mechanistic. It has therefore been challenging to distinguish between different hypotheses that could describe observed patterns. In particular, we evaluated two antigenic seniority mechanisms that have been proposed as explanations for why individuals exhibit raised titres to strains encountered earlier in life [15]: earlier responses could be boosted by subsequent infections, or subsequent responses could be reduced as a result of prior immunity.

There is an apparent discrepancy between our main parameter estimates for antigenic seniority and empirical observations of boosting of antibodies to early infections. Our baseline results (in the absence of waning) suggest that while there is a reduction in the magnitude of response to later infections, the boosting component of antigenic seniority has a negligible effect on observed long-term titres. However, several studies have found evidence for boosting of existing responses following influenza infection [13,14,19–21].

There were two mechanisms in our model that could potentially lead to increased titres to previously encountered strains after subsequent infections. The first was cross-reaction: in our framework, individuals who had been infected with few strains (Fig. 3A) had lower titres than those who had been infected with several strains that are antigenically related (Fig. 3C). We assumed cross-reaction was symmetric in the model, and made the same contribution to titres regardless of infection order.

In contrast, we did not find evidence for boosting of earlier responses as a result of antigenic seniority. In order to investigate this further, we considered the possibility of waning antibodies as a sensitivity analysis. Unfortunately, with cross-sectional data, the boosting and waning processes were not identifiable. Therefore, we assumed a plausible single overall rate of antibody waning and found evidence for boosting as part of an antigenic seniority process. However, although it was possible to force boosting into the model, we suggest that the identifiability issues between boosting and waning and the discrepancy between the model parameters and observed boosting are both consequences of the different timescales on which these immunological processes occur.

Boosting and waning are both likely to contribute to the hierarchal nature of antibody responses to influenza. But while waning of elevated titres has been observed over periods of less than a year [35–38], it is not clear precisely how boosted antibody responses persist over time in the absence of infection [41]. Based on our model results, we suggest that repeated boosting of long-lived antibody responses in the absence of waning is unlikely: such a process would lead to either extremely high titres in older individuals or very low rates of infection, neither of which seem credible. Therefore, in essence, we believe our model provides a plausible description of the acquisition of a stable set of persistent antibodies.

The apparent discrepancies between observed boosting and relatively low titres to historical strains further highlight the need for studies that take repeated measurements of the serological response of individuals against a panel of historical influenza strains [42]. With such data, the mechanistic model presented here could be expanded to explore both the short- and long-term dynamics of influenza immunity. This would help elucidate the precise role of boosting and suppression in antigenic seniority.

We found that cross-reactivity decayed quickly with time, with a half-life of 2.4 y. Hence, there was little cross-reaction between influenza A(H3N2) strains that circulated several years apart. We also considered a model that included broad cross-reactivity between strains, but when we fitted this model to data, the parameter estimate for broad cross-reactivity was zero, indicating that this additional component was not necessary to reproduce observed serological patterns. However, there is evidence that individuals are capable of producing broadly cross-reactive antibodies following infection with a pandemic strain [12,40], and that individuals can exhibit a longitudinal increase in neutralising titres against pandemic strains that are no longer circulating [13]. Again, this highlights the need for longitudinal studies of serological responses against a panel of historical influenza strains. Such data would make it possible to jointly examine the contribution of broad and strain-specific immune responses, and understand how cross-reactive antibodies and antigenic seniority influence observed serological patterns over multiple timescales.

As well as comparing the effects of different immune mechanisms, we estimated infection histories for each individual in our study population. We used this information to measure how frequency of infection varied with age. Although infections became less frequent as individuals progressed through childhood and young adulthood, they occurred at similar rates for individuals above age 30 y (Fig. 4A). It has been suggested that influenza transmission is driven by intense social contacts among younger age groups [43]. The decline in frequency of infection with age may be the result of age-specific differences in social behaviour. A study conducted in the same area of southern China as our serological survey found intense mixing within the under-20-y age groups, which could mean the force of infection was higher within these groups [44]. Unfortunately, we had limited serological data for very young individuals (the youngest participant in the study was aged 7 y); it would be interesting to see how the frequency of infection changes from birth through childhood.

There are some additional limitations to the work described here. We made no prior assumptions about different age groups’ rate of infection, and hence infection history, in the model. An important next step would be to develop an approach that could measure force of infection from cross-sectional data [45]. This could be explored using a model that accounted for population transmission dynamics as well as serological responses. Moreover, we examined serological data from only 151 participants in southern China. It would therefore be helpful to test similar models of serodynamics against observed titres in other populations [42]. We also focused on responses against a panel of A(H3N2) influenza strains. Unlike group 1 influenza viruses such as A(H1N1), A(H1N1p), and A(H2N2), no group 2 viruses other than A(H3N2) have caused a pandemic; it has been suggested that this is why antibody titres specific to HA stalks might be lower for group 2 viruses than for the more antigenically diverse set of group 1 viruses that have circulated in humans [13]. We also do not distinguish between live infection and vaccination in the model; different routes of exposure could influence the process of antigenic sin/seniority in different ways [12,24].

The model we present offers a novel method for simultaneously investigating immune responses and past infections. Studies looking at the antigenic relationship between different influenza strains typically examine cross-reactivity using ferret sera [9,32]. However, the transmission dynamics of influenza is mediated not just by antigenic change in the virus, but also by underlying immunity in the host population. To analyse the evolutionary trajectory of influenza viruses using human sera, it would be necessary to account for past infections, and how these shape the immune response. We propose that a model of serodynamics, as outlined in this paper, would provide the theoretical foundation required to tackle this problem.

Our results also have implications for the analysis of control measures. By considering how a lifetime of infection shapes cross-sectional sera, we have measured the relative importance of different immune mechanisms and past infections in measured serological responses to influenza. As well as influencing the evolution of influenza, such mechanisms could have an impact on the effectiveness of vaccination programmes [7].

We found that the model had limited capacity to accurately predict the magnitude of observed titres to strains that circulated several years before or after the strains to which the model was fitted (S8 Fig.). This is likely the result of the fast decay in cross-reactivity between strains over time. However, the model could generally predict age-specific trends in titres to unencountered strains, even those far from the strain used for fitting. It also reproduced the observed titre levels accurately when fitted strains were close to the test strains in time. This suggests that sufficient representation of past strains, perhaps from every antigenic epoch, would be needed to reproduce all responses accurately. Further, our results were based on neutralisation titres. Similar results are likely to be obtained using HAI, albeit with lower specificity for lower titre values [15,42]. Also, future studies may be able to take advantage of emerging immunological technology based on high throughput protein microarrays [46] and sequence-based measures of B cell diversity [47].

Using a model of cross-sectional serological responses, we have assessed the relative importance of different immune mechanisms and the timing of influenza infection in shaping observed neutralisation titres across the lifetime of an individual. To our knowledge, these two key factors have not previously been combined to fit immunological data. As well as characterising different aspects of the immune response, we have generated individual-level estimates of the frequency and age distribution of influenza infection from cross-sectional serological data. These results demonstrate the value of interpreting immune responses in the context of a lifetime of infection. Integrating the life course of immunity into future analyses of influenza dynamics could therefore lead to a better understanding of population susceptibility and the potential transmissibility of new seasonal strains.

## Supporting Information

S1 Data Dataset and model outputs used to generate figures in main text. (XLS)

S1 Fig Change in standard deviation of residuals of spline fit and individual titres over time. (A) Results from observed data (red line and black points in Fig. 2). (B) Results from model (blue line and grey points in Fig. 2).
(TIFF)

S2 Fig Model residuals. The histogram shows the difference in observed titre across all participants and test strains and the model maximum a posteriori probability estimate.
(TIFF)

S3 Fig Relationship between observed titre against primary infection and total estimated number of infections. If the response to earlier strains was repeatedly boosted after subsequent infections, we might expect to see a positive correlation between the number of infections and titre against the first infecting strain. However, there is little evidence of such a relationship: the Spearman rank correlation coefficient for the two variables is 0.08 (*p* = 0.43).
(TIFF)

S4 Fig Joint posterior estimates for waning, *w*, and antigenic seniority boosting, τ_1_. There is strong evidence of a linear relationship between the two: the Spearman rank correlation coefficient for the two variables is 0.87 (*p* < 0.01).
(TIFF)

S5 Fig Model with broad and specific cross-reactivity. Specific cross-reactivity decays with time, controlled by a parameter σ as in the original model, and strains far apart in time exhibit a fixed broad cross-reactivity, α. Red line, α = 0.1 and σ = 0.3. Blue line, α = 0 and σ = 0.3; hence, there is no broad cross-reactivity, and the model is equivalent to the original framework.
(TIFF)

S6 Fig Schematic of antigenic seniority. (A) Reduced response to subsequent infections using model estimates for individual in Fig. 3B. Bars show the estimated neutralisation titres generated by each infecting strain in Fig. 3B (i.e., contributions from cross-reactive strains are not shown). With each subsequent infection, neutralisation titres are reduced as a result of antigenic seniority. (B) Reduced titres for individual infection history shown in Fig. 3C.
(TIFF)

S7 Fig Sum of absolute model residuals across all strains for each participant. Vertical lines show accuracy of estimates in Fig. 3 compared to other individuals’ estimated titres.
(TIFF)

S8 Fig Prediction of out-of-sample data. For each strain, the model is fitted using data for the other eight strains, then parameter estimates are used to predict titre to the ninth. (A–I) Results for each of the nine test strains. Black points show observed titre against that strain for each participant. Grey points show model predictions. Red line is spline fitted to the data; blue line shows spline fitted to the model predictions, with the 95% confidence interval given by the shaded region.
(TIFF)

S1 Table Parameter estimates for different values of waning, *w*. We assume waning reduces titres to strains in the infection history by a factor *e* ^*−w*^ per year post-infection (details in S1 Text).
(PDF)

S2 Table Parameter estimates for extended model that includes specific and broad cross-reactivity (details in S1 Text). (PDF)

S1 Text Description of model and inference procedure. (PDF)

## Abbreviations:

HA: haemagglutinin
HAI: haemagglutination inhibition
NA: neuraminidase
