# Supplementary figures and images for: Estimating the Life Course of Influenza A(H3N2) Antibody Responses from Cross-Sectional Data
Source: PLoS Biol. 2015 Mar 3;13(3):e1002082. doi: 10.1371/journal.pbio.1002082 (PMC4348415; doi:10.1371/journal.pbio.1002082)

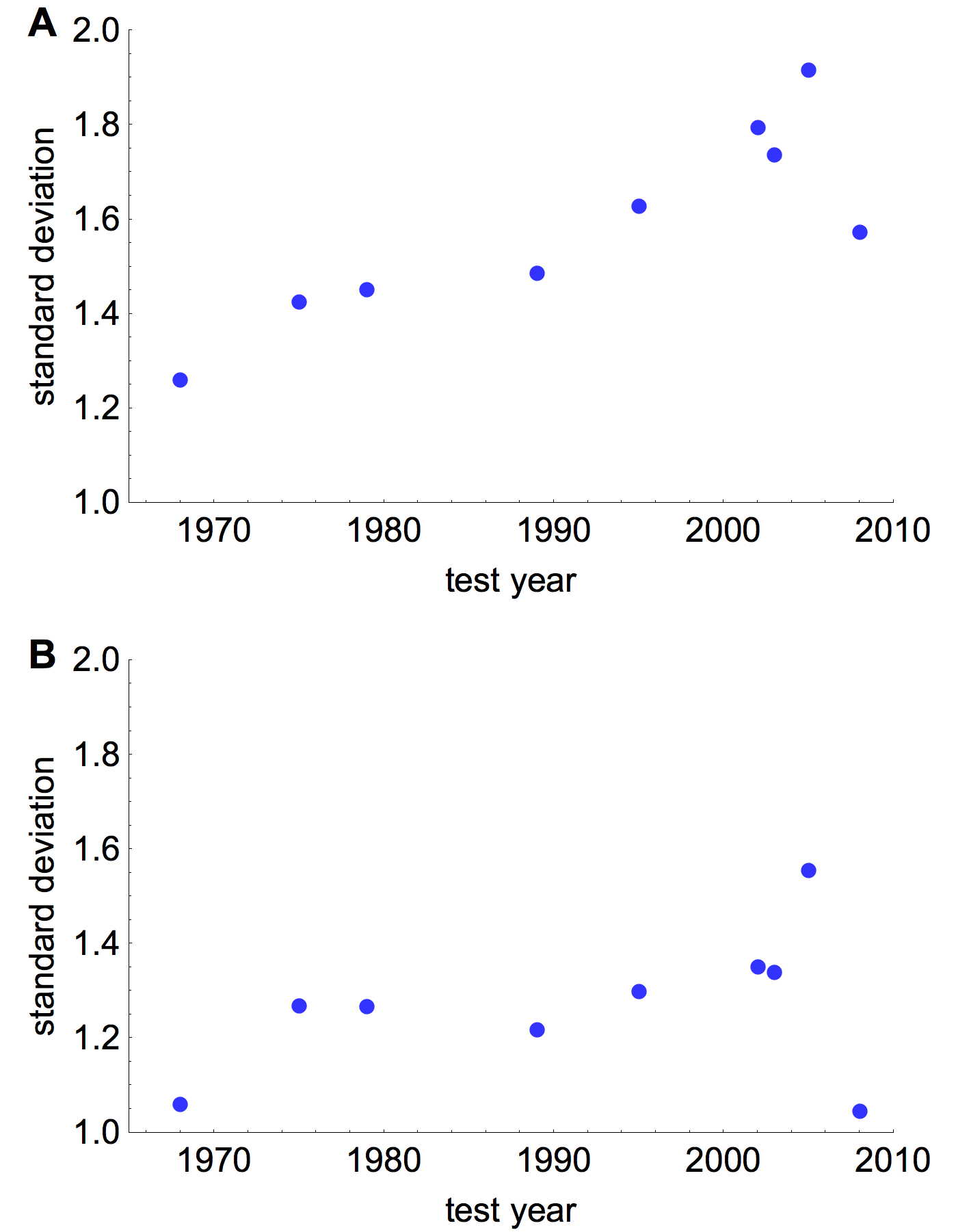

Supplement: S1 Fig — (A) Results from observed data (red line and black points in Fig. 2). (B) Results from model (blue line and grey points in Fig. 2). (TIFF) [file pbio.1002082.s002.tiff]

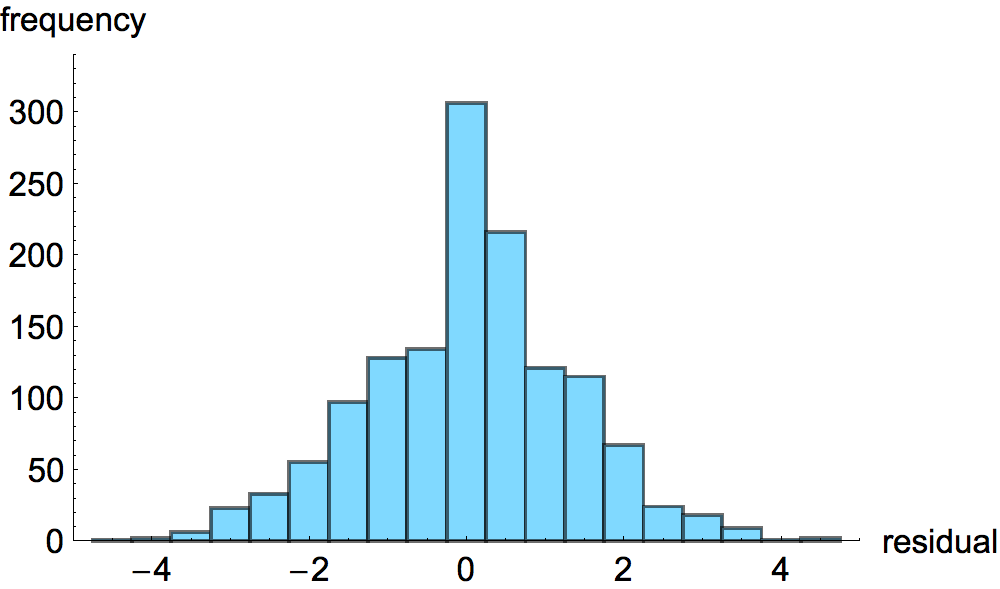

Supplement: S2 Fig — The histogram shows the difference in observed titre across all participants and test strains and the model maximum a posteriori probability estimate. (TIFF) [file pbio.1002082.s003.tiff]

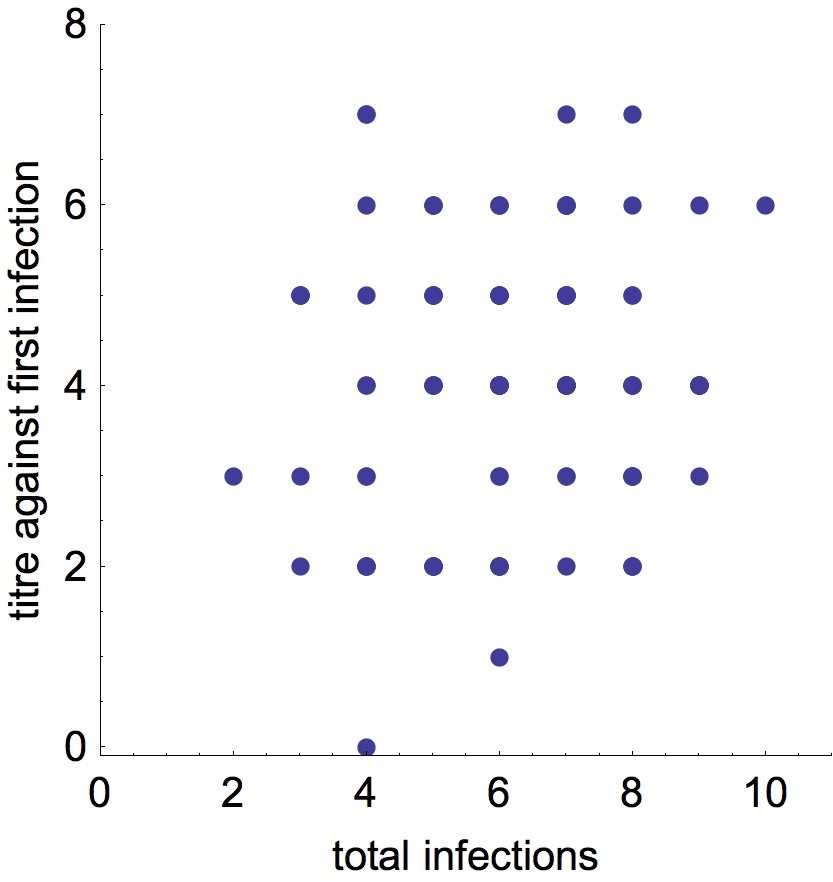

Supplement: S3 Fig — If the response to earlier strains was repeatedly boosted after subsequent infections, we might expect to see a positive correlation between the number of infections and titre against the first infecting strain. However, there is little evidence of such a relationship: the Spearman rank correlation coefficient for the two variables is 0.08 (p = 0.43). (TIFF) [file pbio.1002082.s004.tiff]

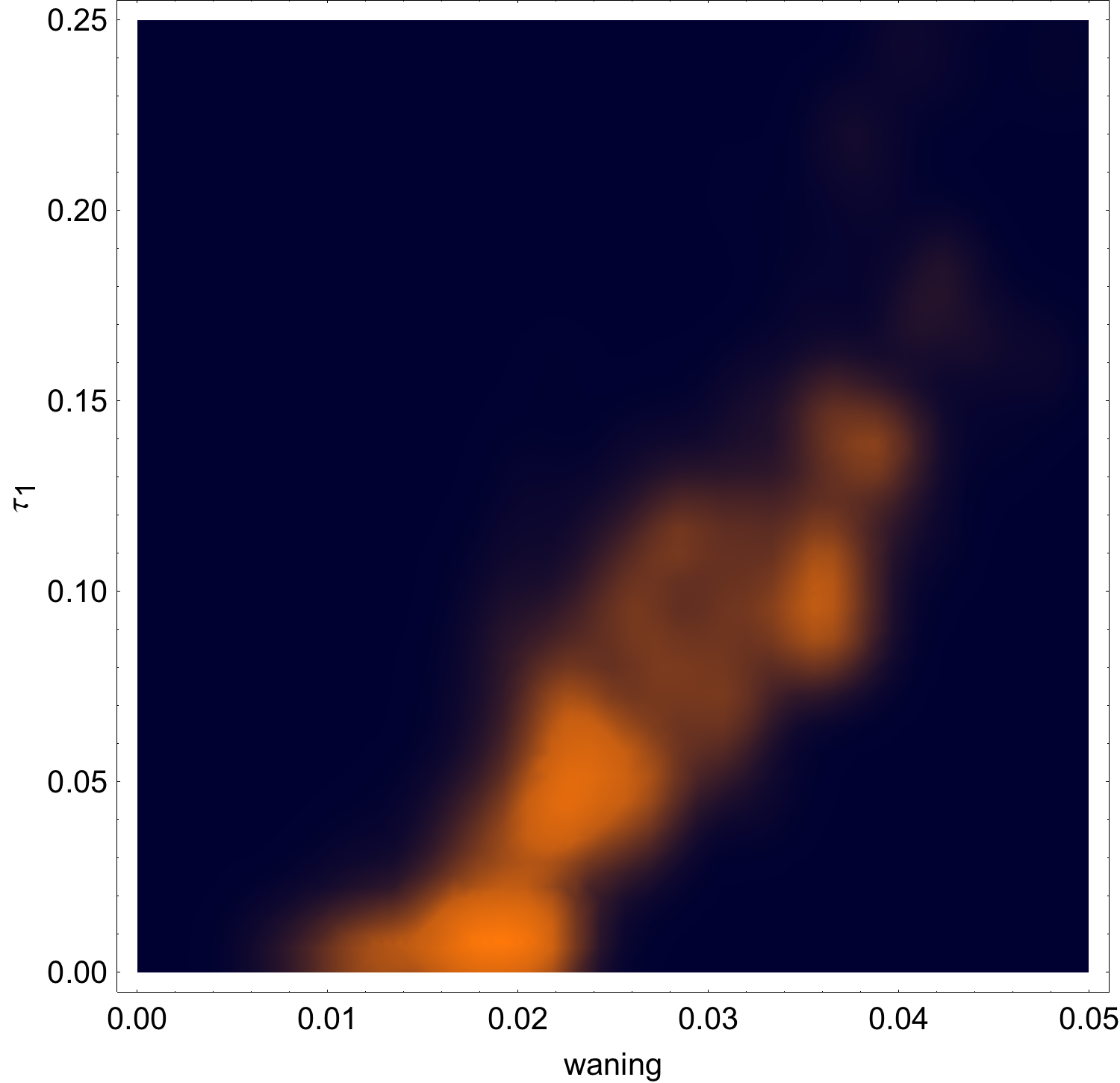

Supplement: S4 Fig — There is strong evidence of a linear relationship between the two: the Spearman rank correlation coefficient for the two variables is 0.87 (p < 0.01). (TIFF) [file pbio.1002082.s005.tiff]

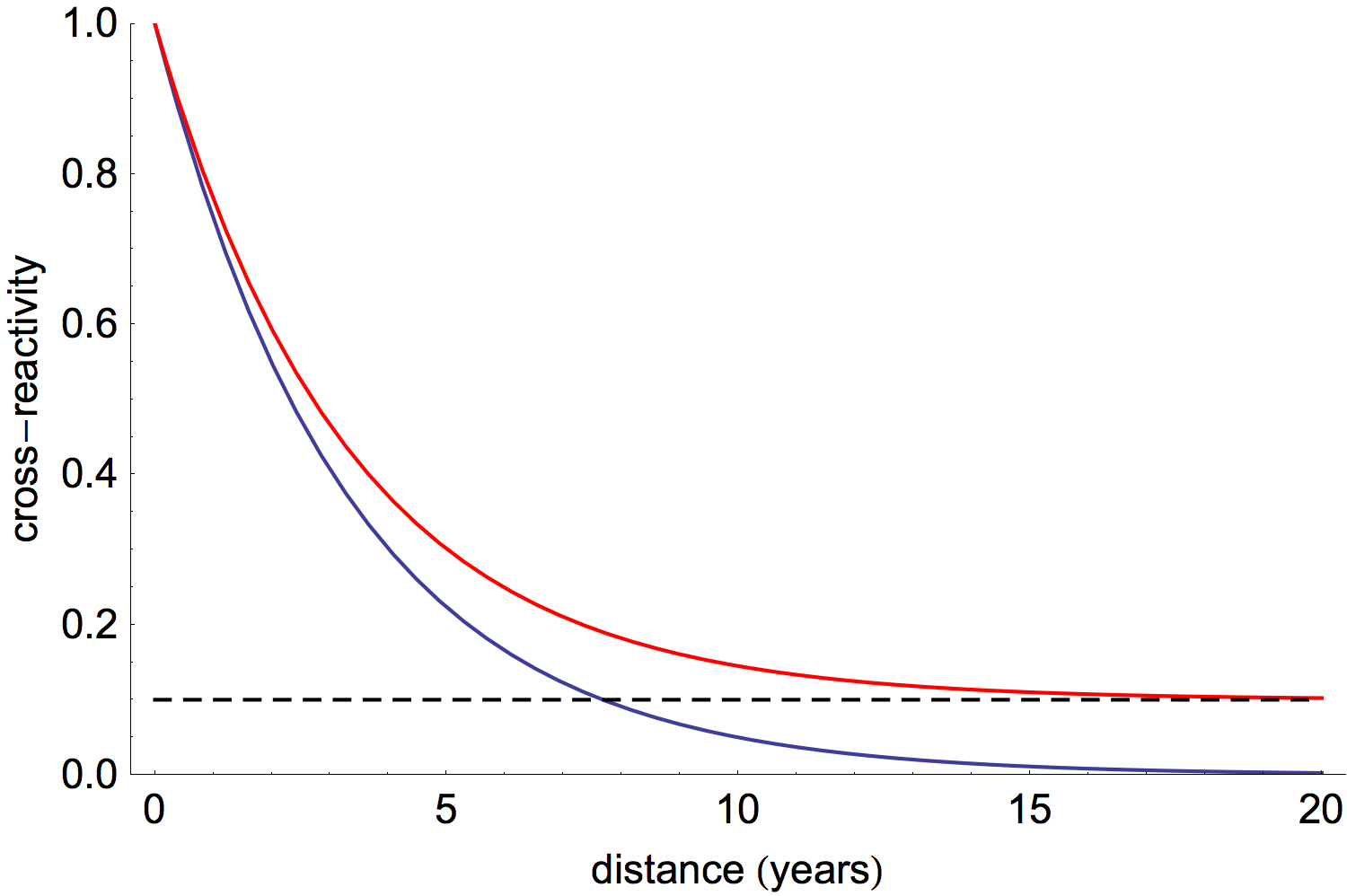

Supplement: S5 Fig — Specific cross-reactivity decays with time, controlled by a parameter σ as in the original model, and strains far apart in time exhibit a fixed broad cross-reactivity, α. Red line, α = 0.1 and σ = 0.3. Blue line, α = 0 and σ = 0.3; hence, there is no broad cross-reactivity, and the model is equivalent to the original framework. (TIFF) [file pbio.1002082.s006.tiff]

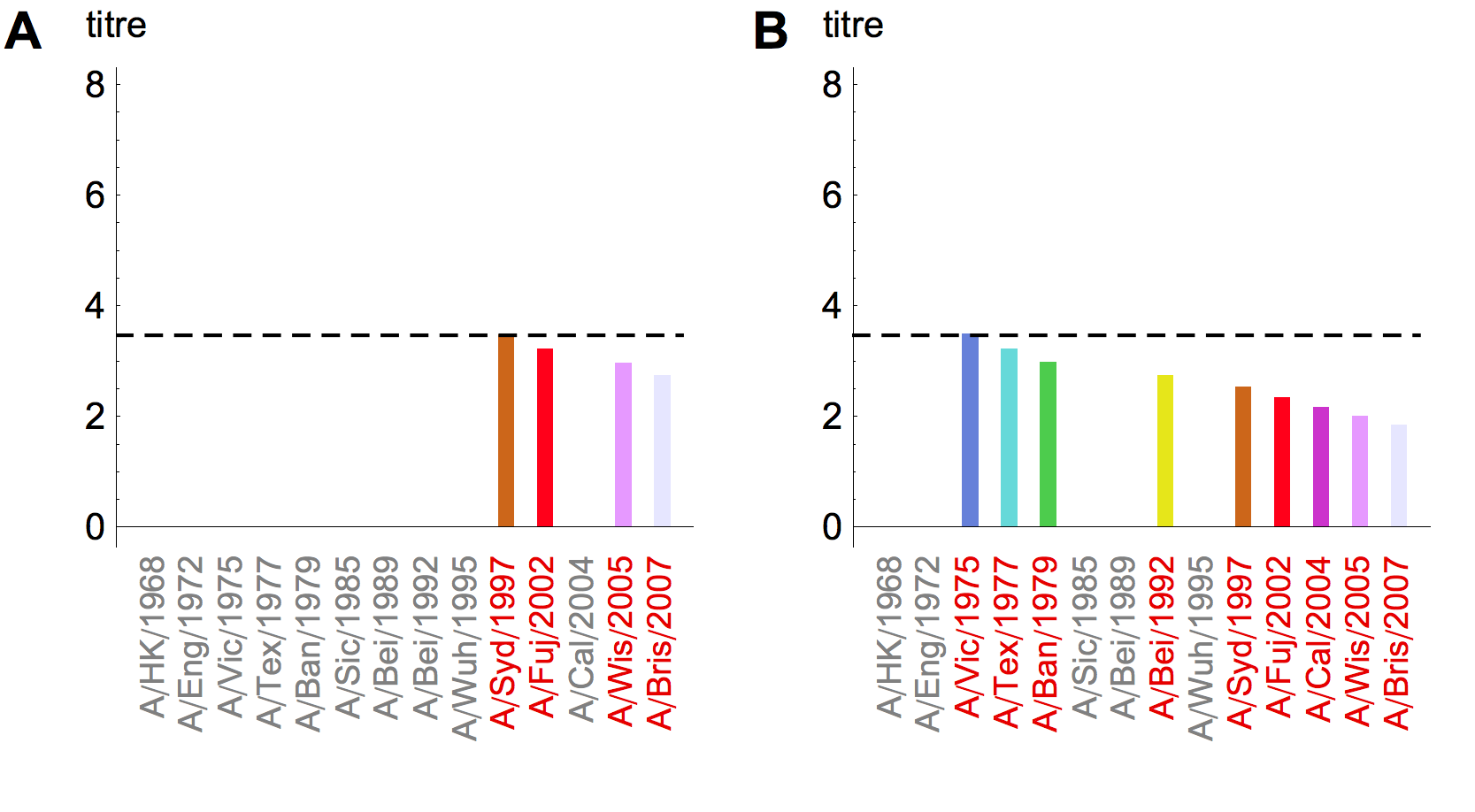

Supplement: S6 Fig — (A) Reduced response to subsequent infections using model estimates for individual in Fig. 3B. Bars show the estimated neutralisation titres generated by each infecting strain in Fig. 3B (i.e., contributions from cross-reactive strains are not shown). With each subsequent infection, neutralisation titres are reduced as a result of antigenic seniority. (B) Reduced titres for individual infection history shown in Fig. 3C. (TIFF) [file pbio.1002082.s007.tiff]

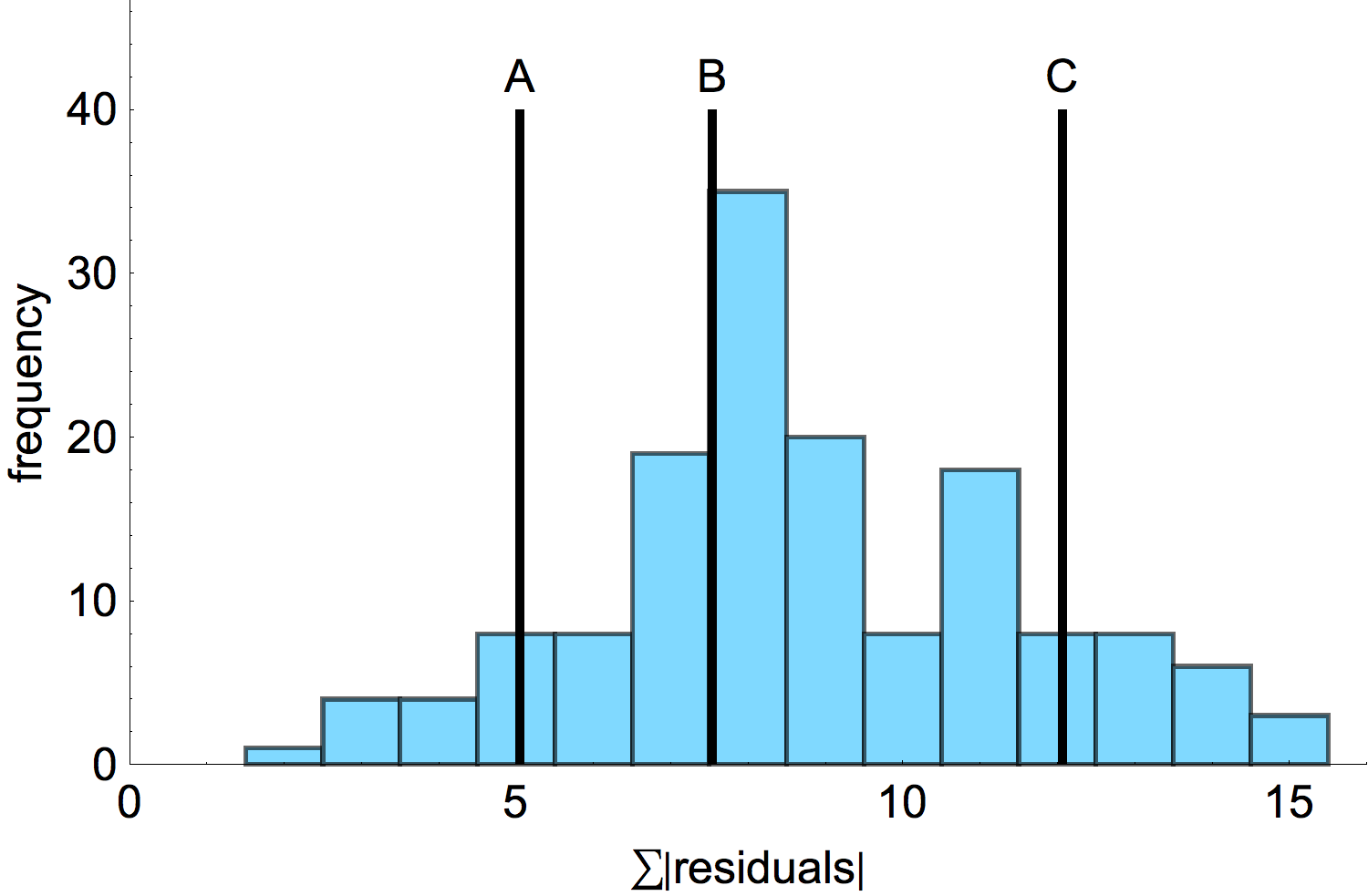

Supplement: S7 Fig — Vertical lines show accuracy of estimates in Fig. 3 compared to other individuals’ estimated titres. (TIFF) [file pbio.1002082.s008.tiff]

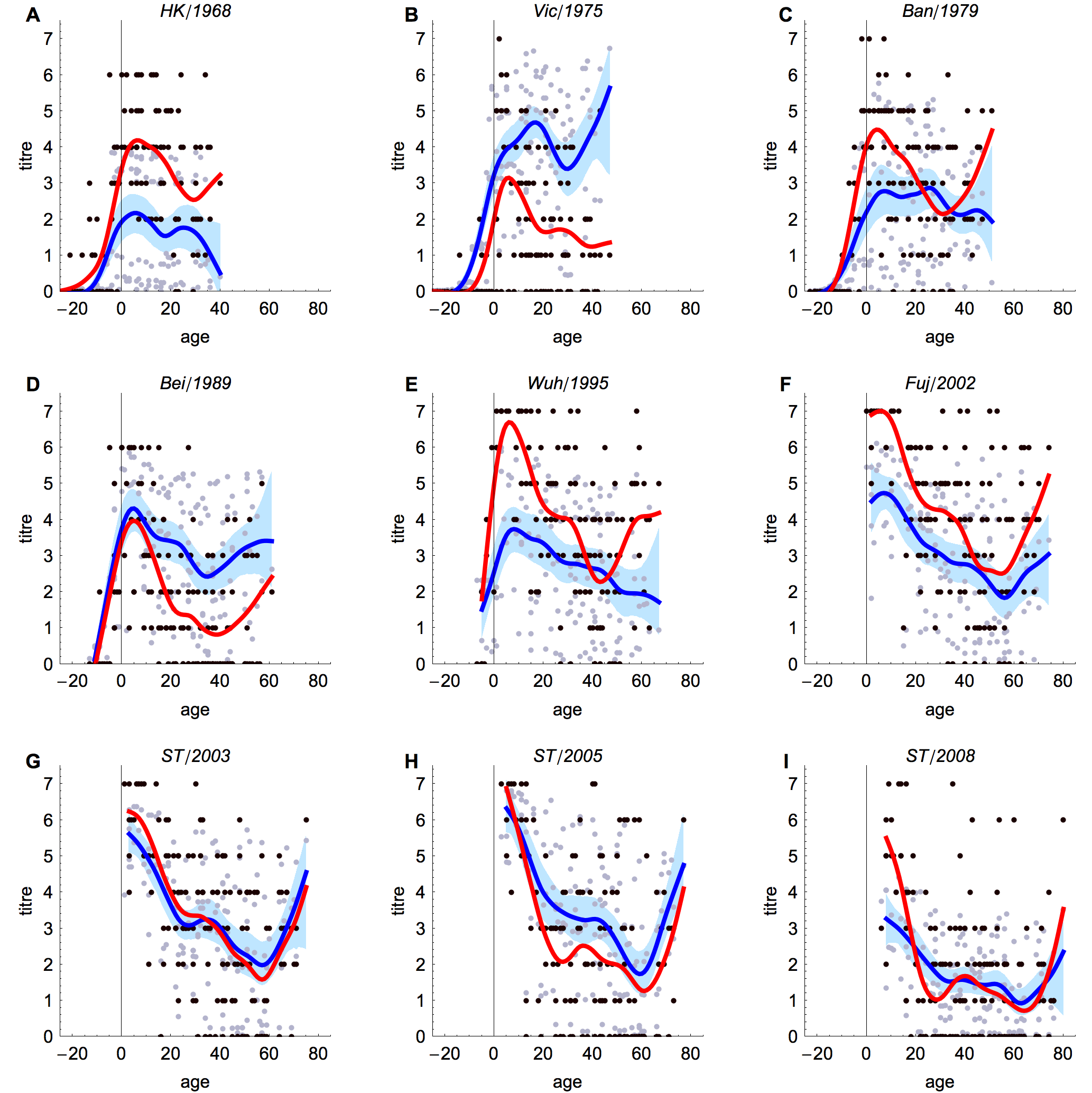

Supplement: S8 Fig — For each strain, the model is fitted using data for the other eight strains, then parameter estimates are used to predict titre to the ninth. (A–I) Results for each of the nine test strains. Black points show observed titre against that strain for each participant. Grey points show model predictions. Red line is spline fitted to the data; blue line shows spline fitted to the model predictions, with the 95% confidence interval given by the shaded region. (TIFF) [file pbio.1002082.s009.tiff]
