# Supplementary material for: Estimating the Life Course of Influenza A(H3N2) Antibody Responses from Cross-Sectional Data
Source: PLoS Biol. 2015 Mar 3;13(3):e1002082. doi: 10.1371/journal.pbio.1002082 (PMC4348415; doi:10.1371/journal.pbio.1002082)
Supplement: S1 Table — We assume waning reduces titres to strains in the infection history by a factor e −w per year post-infection (details in S1 Text). (PDF) [file pbio.1002082.s010.pdf]

Parameter	$w = 0$	$w = 0.01$	$w = 0.1$
μ	3.01 (2.64–3.42)	4.29 (3.12–4.99)	5.01 (4.03–6.33)
ϵ	0.00 (0.00–0.02)	0.01 (0.00–0.03)	0.01 (0.01–0.02)
σ	0.29 (0.25–0.33)	0.29 (0.23–0.33)	0.29 (0.25–0.33)
τ_1	0.00 (0.00–0.01)	0.00 (0.00–0.03)	0.05 (0.03–0.08)
τ_2	0.06 (0.02–0.09)	0.11 (0.08–0.15)	0.13 (0.09–0.17)
