# Supplementary material for: Estimating the Life Course of Influenza A(H3N2) Antibody Responses from Cross-Sectional Data
Source: PLoS Biol. 2015 Mar 3;13(3):e1002082. doi: 10.1371/journal.pbio.1002082 (PMC4348415; doi:10.1371/journal.pbio.1002082)
Supplement: S2 Table — (PDF) [file pbio.1002082.s011.pdf]

| Parameter | Definition | Estimate (95% credible interval) |
|---|---|---|
| $\mu$ | Primary boosting | 3.01 (2.64–3.42) |
| $\epsilon$ | Measurement error | 0.00 (0.00–0.02) |
| $\sigma$ | Specific cross-reactivity | 0.29 (0.25–0.33) |
| $\alpha$ | Broad cross-reactivity | 0.00 (0.00–0.01) |
| $\tau_1$ | Antigenic seniority (boost prior response) | 0.00 (0.00–0.01) |
| $\tau_2$ | Antigenic seniority (suppress new response) | 0.06 (0.02–0.09) |
