# Supplementary material for: Estimating the Life Course of Influenza A(H3N2) Antibody Responses from Cross-Sectional Data
Source: PLoS Biol. 2015 Mar 3;13(3):e1002082. doi: 10.1371/journal.pbio.1002082 (PMC4348415; doi:10.1371/journal.pbio.1002082)
Supplement: S1 Text — (PDF) [file pbio.1002082.s012.pdf]

Supplementary Text S1: Estimating the life course of influenza A(H3N2) antibody responses from cross-sectional data

Adam J. Kucharski[1,2], Justin Lessler[3], Jonathan M. Read[4], Huachen Zhu[5,6], Chao Qiang Jiang[7], Yi Guan[5,6], Derek A. Cummings[3], Steven Riley[2]

[1]Department of Infectious Disease Epidemiology, London School of Hygiene & Tropical Medicine [2]MRC Centre for Outbreak Analysis and Modelling, Department of Infectious Disease Epidemiology, School of Public Health, Imperial College London [3]Department of Epidemiology, Johns Hopkins Bloomberg School of Public Health, Johns Hopkins University [4]Department of Epidemiology and Population Health, Institute of Infection and Global Health, Faculty of Health and Life Sciences, University of Liverpool [5]International Institute of Infection and Immunity, Shantou University Medical College, Shantou, Guangdong, China [6]Department of Microbiology, The University of Hong Kong, Hong Kong SAR, China [7]Guangzhou No. 12 Hospital, Guangzhou, Guangdong, China

Likelihood function

For an individual i who was infected with strains in the set X, we assumed the true titre against strain j titre was Poisson distributed with mean λ_{ij}. Let k denote this true log titre, where $0 \leq k \leq 8$. Hence the probability of having true titre k was as follows (we denote $\lambda_{ij} = \lambda$ for brevity):

$$f(k \mid \theta, X) = \begin{cases} \frac{\lambda^k e^{-\lambda}}{k!} & \text{if } k \neq 8; \\ \sum_{k=8}^{\infty} \frac{\lambda^k e^{-\lambda}}{k!} & \text{else.} \end{cases} \tag{1}$$

We accounted for potential observation error by assuming that there was a uniform probability of observing a titre different to the true one. Hence the likelihood of observing titre c_j against test strain j was equal to the sum over all possible true titres:

$$L(c_j) = \sum_k \mathbb{P}(\text{true titre is } k) \times \mathbb{P}(\text{observe } c_j \mid \text{true titre is } k). \tag{2}$$

We also assumed the following uniform observation model:

$$\mathbb{P}(\text{observe } c_j \mid \text{true titre is } k) = \begin{cases} 1-\varepsilon & \text{if } k = c_j; \\ \varepsilon/8 & \text{else.} \end{cases} \tag{3}$$

The likelihood of observing titre c_j could therefore be calculated by combining Equations 1 and 3:

$$L(c_j \mid \theta, X) = \sum_{k=0}^{8} \mathbb{P}(\text{true titre is } k).\mathbb{P}(\text{observe } c_j \mid \text{true titre is } k) \tag{4}$$

$$= \sum_{k \neq c_j} \frac{\varepsilon}{8} \mathbb{P}(\text{true titre is } k) + (1-\varepsilon)\mathbb{P}(\text{true titre is } c_j) \tag{5}$$

$$= \sum_{k \neq c_j} \frac{\varepsilon}{8} f(k; \theta, X) + (1-\varepsilon) f(c_j; \theta, X) \tag{6}$$

$$= \frac{\varepsilon}{8}[1 - f(c_j; \theta, X)] + (1-\varepsilon) f(c_j; \theta, X) \tag{7}$$

$$= (1 - \frac{9\varepsilon}{8}) f(c_j; \theta, X) + \frac{\varepsilon}{8} \tag{8}$$

Without loss of generality we set $\varepsilon = 8\nu/9$ to get:

$$L(c_j \mid \theta, X) = (1-\nu) f(c_j; \theta, X) + \frac{\nu}{9}\,. \tag{9}$$

Parameter estimation

We fit our model to serological data using Markov chain Monte Carlo [1]. Using the likelihood function in Equation 9, we jointly estimated θ and X for each individual via a Metropolis-Hastings algorithm using 20 million iterations (including a 5 million burn in period). On alternate iterations, we resampled infection histories for each individual (which were independent across individuals), and model parameters (which were shared across all individuals).

To obtain the parameter estimates in Table 1, we calculated the median of the posterior distribution for each parameter, as well as the 95% credible interval. The illustrative plots in Figures 2 and 3 used the maximum likelihood parameter estimates and infection histories. The measured uncertainty in our parameter estimates is shown in Table 1; the variability in estimated infection histories is shown in Figure 4.

Model of specific and broadly cross-reactive responses

To examine whether broadly cross-reactive antibodies might contribute to observed titres, as has previously been observed during influenza infection [2, 3, 4], we extended our model to incorporate a fixed amount of broad cross-reaction between distant strains. In our original formulation, cross-reactivity declined with the time between strains. The level of cross-reaction between a test strain j and infecting strain m was therefore given by $d(j, m) = e^{-\sigma|t_m - t_j|}$, where $|t_m - t_j|$ was the number of years between strains j and m, and σ was a parameter to be fitted. If σ was large, it was equivalent to having no cross-reactivity between strains.

We extended the model to incorporate broad cross-reactivity by assuming that distant strains would still have a degree of cross-reactivity, controlled by a parameter α: $d(j, m) = \alpha + (1 - \alpha)e^{-\sigma|t_m - t_j|}$. As before, σ represented the degree of strain-specific cross-reactivity. After infection with any strain, individuals would therefore have a contribute of α to their titre against any other strain. When $\alpha = 0$, we recovered the original model presented in the main text.

References

[1] Gilks W, Richardson S, Spiegelhalter D (1996) Markov chain Monte Carlo in practice. Chapman & Hall/CRC.

[2] Wrammert J, Koutsonanos D, Li GM, Edupuganti S, Sui J, et al. (2011) Broadly cross-reactive antibodies dominate the human B cell response against 2009 pandemic H1N1 influenza virus infection. J Exp Med 208: 181-93.

[3] Sonoguchi T, Naito H, Hara M, Takeuchi Y, Fukumi H (1985) Cross-subtype protection in humans during sequential, overlapping, and/or concurrent epidemics caused by H3N2 and H1N1 influenza viruses. Journal of Infectious Diseases 151: 81–88.

[4] Pica N, Hai R, Krammer F, Wang TT, Maamary J, et al. (2012) Hemagglutinin stalk antibodies elicited by the 2009 pandemic influenza virus as a mechanism for the extinction of seasonal H1N1 viruses. Proc Natl Acad Sci USA 109: 2573-8.
